# Association between depression in carers and malnutrition in children aged 6 months to 5 years

**DOI:** 10.4102/phcfm.v9i1.1270

**Published:** 2017-01-30

**Authors:** Keneilwe Motlhatlhedi, Vincent Setlhare, Adewale B. Ganiyu, Jacqueline A. Firth

**Affiliations:** 1 Department of Family Medicine and Public Health, University of Botswana, Botswana; 2 Office of HIV/AIDS, United States Agency for International Development, United States

## Abstract

**Background:**

Childhood malnutrition is an important risk factor for child mortality and underlies close to 50% of child deaths worldwide. Previous studies have found an association between maternal depression and child malnutrition, but it is not known whether this association exists in Botswana. In addition, previous studies excluded non-maternal primary caregivers (PCGs). It is unclear whether the association between primary caregiver depression and child malnutrition remains when non-maternal PCGs are included.

**Aim:**

The aim of this study was to determine if there is an association between PCG depression and malnutrition in children aged between 6 months and 5 years in Mahalapye, Botswana.

**Setting:**

The study was conducted in the child welfare clinics of Xhosa and Airstrip clinics, two primary health care facilities in Mahalapye, Botswana.

**Methods:**

This was a case control study. Cases were malnourished children aged between 6 months and 5 years, and controls were non-malnourished children matched for age and gender. The outcome of interest was depression in the PCGs of the cases and controls, which was assessed using the Patient Health Questionnaire 9 (PHQ 9), a depression screening tool.

**Results:**

From a sample of 171 children, 84 of whom were malnourished, we found that the malnourished children were significantly more likely to have depressed PCGs (odds ratio = 4.33; 95% CI: 1.89, 9.89) than non-malnourished children in the 6-month to 5-year age group; the PCGs of malnourished children also had lower educational status.

**Conclusion:**

This study found a significant association between PCG depression and child malnutrition.

## Introduction

Childhood malnutrition is an important risk factor for child mortality and underlies close to 50% of child deaths worldwide.^1,2^ Reducing the prevalence of malnutrition may contribute to the success of child survival strategies.^2^ In order to curb the high prevalence of malnutrition, it is important to identify and address all factors that contribute to poor child nutrition. Depression, one of the most prevalent mental illnesses, is more common in women of child-bearing age,^3^ and maternal depression has been linked to poor child growth outcomes in developing countries.^4,5^ Globally, an estimated 350 million people suffer from depression with higher prevalence in low- and middle-income countries. A South African study estimated depression prevalence rates of 9.7%, while two studies in Botswana found depression prevalence of 25% and 31%. Both studies in Botswana may be an overestimation of the population prevalence of depression in the country as they were done in high HIV prevalence settings and HIV is associated with higher depression levels.^6,7^ Inclusion of mental health screening of PCGs could help develop more efficacious child nutrition programmes and identify children at higher risk of poor outcomes.

Maternal depression has been consistently associated with poor child growth outcomes in South Asia.^8,9,10,11^ This finding is not homogenous in other regions. A study in Brazil found that maternal depression was associated with poor child growth (odds ratio, OR = 1.8),^12^ while another found that the association between maternal depression and child malnutrition as found on crude analysis was diminished by adjusting for socio-economic variables (*p* = 0.319, *p* = 0.934 and *p* = 0.580, for underweight, stunting and wasting, respectively).^13^ In a study conducted in Nigeria, maternal depression was associated with child malnutrition at 3 and 6 months but not at 9 months.^14^ A study in Uganda, which included children aged 1 to 5 years, found that mothers of malnourished children had higher odds of being depressed, adjusted OR = 2.4 (95% CI: 1.11, 5.18).^15^ A South African study of malnourished children aged less than 3 years who were referred to a child psychiatric clinic for evaluation found that 36.8% of the children were diagnosed with a feeding disorder of caregiver–infant reciprocity which was often associated with maternal depressed mood.^16^ It is possible that there was a selection bias in this study as the malnourished children were referred to a psychiatric clinic. Another study in South Africa which investigated the association between post-natal depression and infant growth did not find an association. Although maternal depression at 2 months was associated with weight lower than the 10th percentile at 18 months, *p* = 0.051, this association was not significant after adjusting for birth weight, *p* = 0.13.^17^ Although it did not investigate an association with malnutrition, a South African study of mental well-being of caregivers found that the prevalence of depression was higher in caregivers who were not the birth mothers of children (35.5%) than birth mothers (28.9%).^18^

There were no studies that investigated the association of child nutritional status and depression in caregivers other than the mother. However, it is interesting to note that a study in Botswana found that children cared for by their guardians were at a higher risk of being malnourished; there is paucity of information about the relationship between child nutrition and caregiver depression in Botswana.^19^ The aim of this study was to determine if there is a relationship between malnutrition in children aged between 6 months and 5 years and depression in their primary caregivers (PCGs) in Botswana.

## Research methods and design

### Study design

This was a case control study.

### Settings

This study was conducted in two primary health care clinics (Airstrip and Xhosa clinics) in Mahalapye, Botswana, which has a population of 43 000. Unemployment amongst the economically active population in the district was estimated at 24%, which is higher than the national unemployment rate of 19.9%, and over 50% of the population of Mahalapye are classified as being in the lower wealth statuses according to census criteria. The prevalence of depression in Mahalapye district is unknown. There is one district hospital which receives referrals from the surrounding clinics and health posts. All the health facilities have child welfare clinics (CWCs) which provide child welfare services for children younger than 5 years. These services include growth monitoring, immunisations and provision of supplementary foods.

### Study population and sampling strategy

The sample population were all children aged between 6 months and 5 years who presented at the CWCs of the study sites with their PCGs. We included PCGs who resided in the catchment area of the study sites and were fluent in English or Setswana. All children with an acute illness or a chronic medical condition (including HIV infection) were excluded.

Assuming a prevalence of depression of 20% in the cases and 10% in the controls, the final enrolment of 171 participants was more than the necessary sample size to maintain a power of 80% and 95% confidence level. The expected prevalence rates of depression in the study population were based on previous findings of depression prevalence ranging from 5% to 15% in the southern African region but higher prevalence of depression in high HIV prevalence settings such as Botswana.^6,7^

The sampling strategy was convenience sampling with the cases enrolled first. The control for each malnourished child was the first well-nourished child matched for age and gender as well as caregiver age.

### Data collection

Data collection was done over a 5-month period from March to July 2015. Each clinic had a research assistant who was trained by the lead researcher. The children’s weight was measured on Salter^®^ hanging scales, with each child fully undressed. Their heights were measured on a Seca^®^ stadiometer for those over 2 years and measuring boards for those aged less than 2 years. All children with anthropometric measurements (weight-for-age, weight-for-height or height-for-age) below -2 z-scores on the World Health Organization growth charts were considered to be malnourished, and were identified as cases. Children with z-score above -2 were considered not to be malnourished. After enrolling cases, each case was matched to a child without moderate or severe malnutrition by gender and age as well as PCG age. Once a child was identified as malnourished or as a match, the research assistant would ascertain if the person who had brought the child to the clinic was the PCG using a set of predetermined questions. If a child lived with its biological mother, the mother was considered the PCG. The research assistant would then obtain informed consent from the PCG, then collect their baseline characteristics and determine their depression status using the PHQ 9, with all scores above 10 classified as cases of depression. The questionnaire was translated to Setswana by the lead researcher then back translated to English. The research assistant explained the instructions then read out the PHQ 9 in a language that the PCG preferred. The research assistant entered the PCGs answers onto a study pro forma. All those found to have a score of 10 and above were referred to the district hospital psychology clinic for more formal assessment, according to an agreement made with the psychology department before the commencement of the study. After all the cases had been enrolled, control matching for each case was done based on age and gender of the child and age of the PCG.

The PHQ 9 is a depression screening tool that has been validated in primary health care settings in North America^20^ as well as Kenya^21^ and South Africa.^22^ It has a sensitivity of 74% and a specificity of 91% at a cut-off score of 10.^20^ Both the baseline characteristics questionnaire and the PHQ 9 were piloted on 10 child–caregiver pairs at Madiba clinic, a smaller clinic in Mahalapye, which serves a community similar to the ones served by the two study sites.

### Data analysis

Data were entered in the statistical software program, SPSS^®^ (Version 23, Released 2015. Armonk, NY: IBM Corp). Descriptive data were analysed as proportions for categorical data, including gender of the children and of the PCGs, and means with standard deviation for continuous data, including ages of the children and of the PCGs. Odds ratios with 95% confidence interval were calculated to determine if there was an association between child nutrition and maternal depression.

## Ethical considerations

The study protocol was approved by the University of Botswana IRB (IRB number 116), the Ministry of Health HRU (ref no. PPME 13/18/1 V (337)) and the Mahalapye District Hospital ethics review committee (ref no. MH/DHMT/1/7/7). Permission to conduct the study was sought from the heads of each clinic. The staff at the CWCs of each clinic were informed about the study, and the study was explained to them.

## Results

**FIGURE 1:**
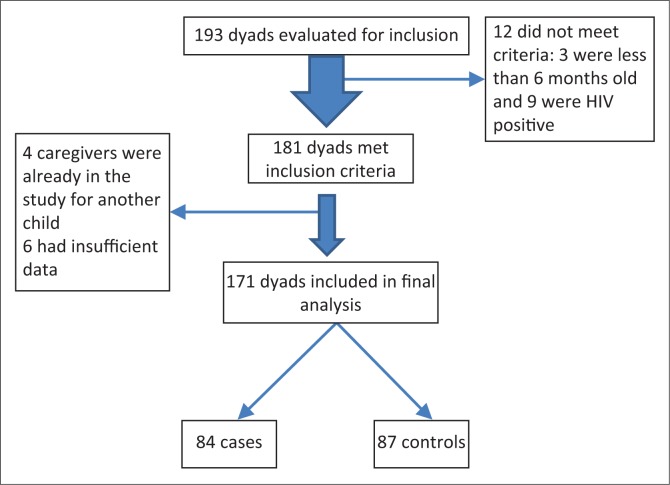
Flowchart showing study participant recruitment.

There were 193 caregiver–child dyads that were initially evaluated for enrolment into the study; however, 22 were excluded from the study for various reasons (see Figure 1 for details).

The final sample size was 171 children and caregivers enrolled in the study, of which 84 were cases with the mean age of 33 months (s.d. ± 14.3) and 87 were controls with the mean age of 32 months (s.d. ± 14.9). Fifty per cent of the cases and fifty-five per cent of the controls were females. Table 1 shows the baseline characteristics of the study participants.

**TABLE 1:** Baseline characteristics of the cases and controls.

Variable	Malnourished *N* = 84 (% or s.d.)	Well-nourished *N* = 87 (% or s.d.)
Child’s age in months	33.04 (±14.3)	32.23 (±14.9)
Gender of children		
Female	42 (50.0%)	48 (55.2%)
Male	42 (50.0%)	39 (44.8%)
Primary caregiver’s age in years	34.3 (±11.2)	34.38 (±11.2)
Primary caregiver’s gender		
Female	80 (95.2%)	86 (98.9%)
Male	4 (4.8%)	1 (1.1%)
Educational level of the primary caregiver		
Primary or less	26 (31.0%)	19 (21.8%)
Secondary school	53 (63.1%)	62 (59.8%)
Tertiary	5 (6.0%)	16 (18.4%)

s.d., standard deviation.

The mean ages of the primary care givers were similar, that is, 34 years (s.d. ± 11.2). The majority of PCGs in both groups (80/84 cases and 86/87 controls) were females. Only 5/84 PCGs of cases had completed tertiary school compared with 16/87 of PCGs of controls.

Table 2 below shows that a higher proportion (67.9%) of the cases were either HIV exposed (31/84) or their HIV exposure was unknown (25/84) compared with the controls (27/87 and 13/87, respectively, *p* = 0.004). Most of the PCGs in both groups were unemployed (72/84 cases and 72/87 controls, *p* = 0.229).

**TABLE 2:** HIV exposure, HIV status and PCG characteristics of malnourished and non-malnourished children.

Variable measured	Malnourished *N* = 84 (%)	Well-nourished *N* = 87 (%)	*p*
HIV exposure of the children			
Exposed	31 (36.9)	27 (31.0)	0.004
Not exposed	25 (29.8)	47 (54.0)	
Unknown	26 (31.0)	13 (14.9)	
Missing data	2 (2.4)	0 (0.0)	
HIV status of the children			
Negative	33 (39.3)	55 (63.2)	0.002
Unknown	51 (60.7)	32 (36.8)	
Relationship of primary caregiver to child			
Mother	58 (69.0)	58 (66.7)	0.122
Other	26 (31)	29 (33.3)	
Employment status of the primary caregiver			
Employed	10 (11.9)	15 (17.2)	0.229
Unemployed	72 (85.7)	72 (82.8)	
Other	2 (2.4)	0 (0.0)	
Depression in the primary caregiver			
Yes	28 (33.3)	9 (10.3)	0.001
No	56 (66.7)	78 (89.7)	

PCG, primary caregivers.

The depression status of the two groups were also different with 28/84 PCGs in the case group diagnosed as depressed compared with 9/87 of the controls. Table 2 compares the difference in characteristics of the cases and controls in terms of HIV exposure and status and PCG characteristics

Depression in the PCGs was significantly associated with malnutrition in the child, OR= 4.33 (95% CI: 1.90, 9.90), *p* = 0.001. Having a PCG without tertiary education also increased the probability of a child being malnourished, OR= 3.56 (95% CI: 1.24, 10.28), *p* = 0.018. The monthly income of the PCG was not significantly related to the child’s nutritional status. Table 3 shows the bivariate analysis with malnutrition as the dependent variable, while PCG depression, income, educational attainment and child HIV status were the independent variables.

**TABLE 3:** Bivariate analysis of the relationship between child malnutrition and depression and possible confounding variables.

Co-variate	Odds ratio	*p*
**PCG depression**		
Not depressed	1	
Depressed	4.33 (1.90–9.90)	0.001
**HIV status of child**		
Unknown	1	
Negative	0.37 (0.20–0.70)	0.002
**Relationship of child to PCG**		
Mother	1	
Other	1.12 (0.59–2.12)	0.44
**PCG education**		
Tertiary	1	
Less than tertiary	3.56 (1.24–10.28)	0.02
**Income of primary caregiver**		
More than P1000/month	1	
Less than P1000/month	1.73 (0.76–3.92)	0.19

PCG, primary caregivers.

## Discussion

The major finding of this study is that there is a statistically significant relationship between malnutrition in children aged between 6 months and 5 years and depression in their PCGs in Mahalapye (OR 4.33; 95% CI: 1.90, 9.90, *p* = 0.001). Similar findings were reported in South Africa where depressed maternal mood was reported in 63 out of 179 caregivers of children with failure to thrive.^23^ In a Nigerian case control study, maternal depression was more likely to be associated with child malnutrition, OR = 4.21 (95% CI: 1.36, 13.20) and OR = 3.34 (95% CI: 1.18, 9.44) for weight and height, respectively, below 5th percentile at 6 months,^14^ and in South India where the OR for malnutrition in children whose mothers had postpartum depression was 7.8 (95% CI: 1.6, 38.51).^10^ A cross-sectional study in Brazil^12^ found an association between depressive symptoms in the mother and stunting in their children, reported to be 1.8 (95% CI: 1.1, 2.9).

Our findings differed from a study in South Africa, where researchers did not find a statistically significant association between maternal depression and child weight or height below the 10th percentile.^17^ This difference may be because the South African study focused on a younger age group, 2 to 18 months. The effects of maternal (or PCG) depression on child nutrition may not manifest until later, as shown in South India, a region with high child malnutrition rates. A study there found that postpartum depression was associated with poor child growth (OR 7.4; 95% CI: 1.6, 38.5), whereas there was not a significant association with current major depression (OR 3.1; 95% CI: 0.9, 9.7).^10^ A Brazilian cohort study which followed up children from birth till 4 years found an association on crude analysis which became insignificant after adjusting for socio-economic variables.^13^ However, in our study, there was no statistically significant difference between child nutrition and monthly income (*p* = 0.19) for the two groups. Factors other than socio-economic status may be more significant in our setting, PCG mental health being one of them.

Depression is characterised by apathy and low energy levels which may affect the ability of a caregiver to identify and adequately respond to the cues of a child.^5,24^ The disease may also affect the ability of a PCG to implement strategies to prevent child illness like hand washing and other good hygiene practices; infants of depressed mothers had higher odds of having more frequent diarrhoeal episodes than infants of non-depressed mothers in Nigeria^14^ and Pakistan.^11^ It has also been found that depressed mothers tend to breastfeed for a shorter duration.^25^ Our sample size was not sufficient to analyse the children in different age groups; it is, however, possible that malnutrition secondary to poor feeding practices and weaning was more prevalent in infancy. The effect of weaning may overshadow the effect of PCG depression and this may account for the findings of previous studies which included younger age groups only and found no relationship between malnutrition in the children and depression in their PCGs.

These findings and the findings of two other studies which found that postpartum depression was associated with poor infant growth at 6 months^26^ and at 12 months^11^ suggest that maternal depression precedes poor child growth; it is also possible that a PCG becomes depressed as a result of the child not growing well, especially if the child was also unwell.

We also found that lower PCG education was associated with an increased probability of child malnutrition, *p* = 0.02. Anoop et al. found that low maternal intelligence was associated with a 4-fold increase in the probability of malnutrition.^10^ It has been found that increasing the educational attainments of women results in better child survival.^27^ PCGs with higher education attainments may make better nutritional choices than less educated PCGs; they may also have a better understanding of child health recommendations. Women with higher educational attainments may be more empowered, financially and even economically, to make decisions in the family that positively impact child nutrition.^25^ It has been suggested that maternal (or PCG) education may also serve as a protective factor against depression and its effects on child care.^4^

The relationship of the child to the PCG had no influence on the child’s nutritional status in this study, *p* = 0.44; however, a previous study in Botswana found that malnourished children had a higher probability of being raised by a guardian, OR 5.67 (1.30–24.73).^19^ Furthermore, in South Africa, the prevalence of depression was found to be higher in caregivers who were not the biological mothers of the children in their care.^18^ It is probable that since most of the children in this study were cared for by their mothers, the study sample was too small to detect a difference. Further studies investigating the relationship between non-maternal caregivers and the nutritional status of children in their care are needed.

It has been proposed that interventions aimed at improving maternal (or PCG) mental well-being could result in better child growth outcomes.^5,24^ This is because preventive strategies, such as hygiene practices, immunisation and health-seeking, are dependent on the PCG being functional and able to understand and implement them. Focusing on PCG to child interactions at primary care level may help identify children who need intervention at an early stage and reduce poor child nutrition outcomes.^24^ We propose that training primary care workers to screen PCGs of children with mild to moderate malnutrition may help decrease the progression to severe malnutrition and also contribute to an improvement in the child’s nutritional status and general health. Community-level interventions and interventions aimed at empowering women may be effective.^24^

The limitations of our study include the inability to determine the causality of the association between child malnutrition and caregiver depression owing to the case control design. Another limitation is that we measured weight-for-age at one point in time instead of determining trends, which may have led to incorrect assessment of child nutritional status. In addition, this study was not powered to assess the association between caregiver depression and different levels of malnutrition. The use of convenience sampling may have led to a sampling bias, which could have been overcome by randomisation of enrolment over a larger geographic area and a longer time frame. The study did not assess the effect of other variables which may affect child nutrition, such as food intake, quality of food, and breastfeeding history. Financial constraints limited the study to only two clinics in Mahalapye, resulting in a study population that is relatively small when compared with the under-5 population of Botswana, thus limiting the generalisability of the study.

## Conclusion

Our study revealed a statistically significant relationship between malnutrition in children aged between 6 months and 5 years and depression in their primary caregivers in the study population.

### Recommendation

Based on the findings of our study, we recommend further studies to investigate 1) the relationship between the mental health of non-maternal PCGs and child malnutrition, 2) the strength of the association between caregiver depression and different levels of malnutrition, 3) causality of the relationship between caregiver depression and child malnutrition (a randomised prospective study) and 4) other potentially contributing factors not assessed by this study, including specific food intake, quality of food and breastfeeding history. More urgently, we recommend that the mental health of PCGs be incorporated into child survival strategies in order to optimally address factors contributing to poor child health outcomes in Botswana.
